# Combined Neutron and X-Ray Diffraction Study of Ibuprofen and Atenolol Adsorption in Zeolite Y

**DOI:** 10.3390/molecules31020384

**Published:** 2026-01-22

**Authors:** Annalisa Martucci, Maura Mancinelli, Tatiana Chenet, Luca Adami, Caterina D’anna, Emmanuelle Suard, Luisa Pasti

**Affiliations:** 1 Department of Physics and Earth Sciences, University of Ferrara, Via Saragat 1, I-44121 Ferrara, Italyluca.adami@unife.it (L.A.); 2 Department of Environmental and Prevention Sciences, University of Ferrara, Via L. Borsari 46, I-44121 Ferrara, Italy; tatiana.chenet@unife.it (T.C.); caterina.danna@unife.it (C.D.); luisa.pasti@unife.it (L.P.); 3 Institute Max von Laue and Paul Langevin, D2B Beamline, BP156, 38042 Grenoble, France; suard@ill.fr

**Keywords:** neutron diffraction, zeolite Y, ibuprofen, atenolol, host–guest interactions, framework flexibility

## Abstract

The widespread occurrence of pharmaceutical residues in aquatic environments necessitates the development of advanced porous materials for efficient remediation. This study investigates the adsorption mechanisms of ibuprofen and atenolol within the high-silica zeolite Y. Batch adsorption experiments demonstrated significant uptake, with loading capacities of 191.6 mg/g for ibuprofen and 273.0 mg/g for atenolol, confirming the material’s effectiveness. Using a combination of neutron and X-ray powder diffraction, complemented by Rietveld refinement and simulated annealing algorithms, we achieved the exact localization of the guest molecules. While the pristine zeolite maintains cubic symmetry $Fd\bar{3}$, the incorporation of pharmaceutical molecules induces significant residual nuclear density and anisotropic lattice distortions. To accurately model these perturbations, a systematic symmetry reduction to the acentric triclinic space group *F*1 was implemented. This approach enabled an ab initio refinement of the structure, revealing that drug uptake of each guest is governed by distinct chemical drivers. Ibuprofen is stabilized via steric confinement and long-range dispersive interactions. In contrast, atenolol stability is governed by electrostatic charge compensation within the zeolitic voids. Our results suggest that the final adsorption geometry is dictated by the spatial orientation of functional groups and host–guest proximity rather than molecular chirality. These results provide a microscopic model describing the fundamental host–guest interactions in FAU zeolites. This structural understanding is an essential step towards the potential use of zeolitic materials in environmental remediation and complex guest sequestration.

## 1. Introduction

Over the past two decades, the anthropogenic discharge of pharmaceutical compounds has been recognized as a primary driver of emerging contamination within aquatic ecosystems. These bioactive molecules are extensively used in human and veterinary medicine, as well as in intensive agricultural and aquacultural practices [1]. However, the frequent application and widespread overconsumption of these substances have resulted in their ubiquitous presence in the environment. In the European Union alone, the therapeutic pharmacopeia includes approximately 3000 distinct substances, ranging from neuroactive agents and antibiotics to lipid regulators [2]. Since conventional wastewater treatment plants (WWTPs) are not specifically designed to sequester complex organic pharmaceuticals, these recalcitrant pollutants often bypass standard treatment processes. Consequently, they migrate through sewage systems and eventually contaminate surface waters, groundwater aquifers, and potable water reservoirs [3]. Recent global assessments of measured environmental concentrations indicate the presence of over 600 pharmaceutical substances in water samples worldwide, with concentrations spanning the range from nanograms to milligrams per liter [4].

Among the myriad of pharmaceutical contaminants, ibuprofen and atenolol are increasingly prevalent in aquatic environments, raising significant ecological and public health concerns. Ibuprofen, a ubiquitous non-steroidal anti-inflammatory drug, is frequently documented in surface waters with concentrations ranging from 0.98 to 280 µg/L, while influent levels can reach up to 600 µg/L. Atenolol, a β-blocker commonly prescribed for cardiovascular conditions, exhibits low biodegradability and similar environmental persistence. Concentrations in surface waters range from 45 to 120 ng/L, while treated effluents frequently exceed 2 µg/L [5,6,7,8].

The widespread occurrence of these micropollutants highlights the limitations of conventional activated sludge processes, which are often insufficient for the complete mineralization of recalcitrant synthetic organics. This technological gap necessitates the development of advanced remediation strategies, such as adsorption onto microporous materials, which offers operational simplicity and broad applicability.

Zeolites, microporous aluminosilicates, have emerged as premier sorbents due to their regular pore architecture, tunable chemical composition, and high ion-exchange capacity [9]. Among these, high-silica zeolite Y (FAU-type) is particularly suitable for the removal of bulky organic contaminants; its large supercages (α-cages), with a diameter of approximately 11.2 Å, provide sufficient internal volume to accommodate complex pharmaceutical molecules like ibuprofen and atenolol. Recent studies have demonstrated the high efficiency of various framework types, such as FAU and BEA, in sequestering diverse organic pollutants from aqueous phases [10,11,12,13,14]. In these systems, adsorption performance is typically governed by framework topology, Si/Al ratio, and the nature of extra-framework cations.

Nevertheless, achieving a rigorous molecular characterization of the interactions between the host and guest molecules under hydrated conditions remains a significant challenge. Despite extensive experimental and computational studies, most investigations focus on macroscopic uptake measurements, batch adsorption isotherms, or indirect spectroscopic probes. Consequently, the precise molecular arrangement of pharmaceutical molecules within hydrated zeolitic pores—including the specific role of co-adsorbed water in stabilizing these species—remains largely speculative.

The lack of direct experimental evidence regarding hydrogen-bonding networks, protonation states, and framework–guest cooperativity under realistic aqueous conditions represents a significant barrier to the transition from empirical screening to the rational, structure-based design of advanced adsorbents.

In this context, neutron powder diffraction (NPD) offers a unique advantage: its exceptional sensitivity to light atoms, particularly hydrogen, enables the direct visualization of both pharmaceutical molecules and co-adsorbed water within zeolitic pores. By providing a high-resolution atomistic description of guest sequestration in high-silica zeolite Y, this work moves beyond macroscopic observations. Building on recent NPD investigations of hydrated amino acids in zeolites [15], the present study aims to provide a high-resolution atomistic description of ibuprofen and atenolol uptake in high-silica zeolite Y. The primary innovation of this work lies in the unambiguous experimental determination of the spatial arrangement and stabilization mechanisms of complex pharmaceuticals within a hydrated zeolitic environment. By contrasting the behavior of a neutral amphiphilic drug (ibuprofen) with a protonated polar molecule (atenolol), we resolve the competitive role of co-adsorbed water and framework interactions. Specifically, this research pursues the following objectives:

- To resolve the precise location and orientation of drug molecules and co-adsorbed water clusters within the zeolitic supercages;
- To identify the protonation states and hydrogen-bonding networks that anchor the pharmaceuticals under realistic hydrated conditions;
- To evaluate the cooperative structural response of the zeolite framework, specifically investigating ordered symmetry reduction induced by guest inclusion;
- To contrast the stabilization mechanisms (steric confinement vs. electrostatic charge compensation) to provide a mechanistic bridge between microscopic structure and macroscopic adsorption performance.

These molecular insights provide a crucial bridge between macroscopic performance and microscopic mechanisms, supporting the rational design of sustainable zeolitic adsorbents for selective environmental remediation.

## 2. Results and Discussion

### 2.1. Structure of Pristine Zeolite Y and Host–Guest Interactions with Water Molecules

Rietveld refinement of the pristine zeolite Y (SiO_2_/Al_2_O_3_ = 30) confirmed a cubic symmetry ($Fd\bar{3}$ space group), with a refined unit cell parameter a = 24.291(1) Å and a corresponding volume of 14,332.7(5) Å^3^ (Table 1).

The framework architecture is defined by two distinct tetrahedral sites (T1 and T2), exhibiting T–O bond distances between 1.600(4) and 1.617(5) Å for T1, and 1.593(4) to 1.603(4) Å for T2.

The T–O–T angles vary significantly (129.3(1)° to 163.7(1)°), indicating localized structural distortions, which are particularly pronounced within the 12-membered ring (12MR) channels (Tables S1 and S2).

**Figure 1:**
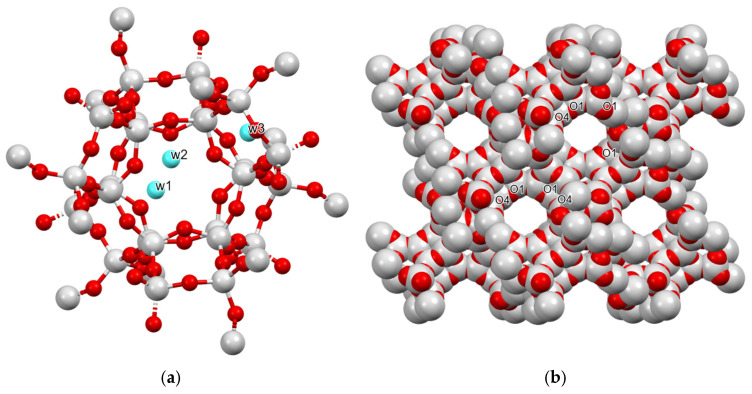
(**a**) 3D framework view of pristine zeolite Y (grey: Si/Al; red: O) showing extra-framework water molecules (light blue spheres: w1, w2, and w3) in the 12MR channels (**b**) 12MR cross-section highlighting framework oxygen sites (O1 and O4) defining channel geometry.

The 12MR window aperture is defined by O···O distances of 9.59 Å (O1–O1) and 11.16 Å (O4–O4), resulting in a calculated ellipticity ε = 1.23. This deviation from ideal circularity is intrinsically correlated with the specific spatial distribution of extra-framework water molecules. Within the supercage, three distinct water sites (w1, w2, and w3) were localized, forming hydrogen-bonded oligomeric chains. The stability of these clusters is governed by short-range intermolecular interactions, notably the w1···w3 and w1···w1 distances, which were refined to 2.475 Å and 2.476 Å, respectively (Figure 1).

The interaction between the framework and the guest molecules is primarily mediated by the O1 oxygen site. The O1···w3 distance of 3.077 Å is indicative of a weak hydrogen bond, which nonetheless induces a measurable contraction of the T–O–T angle at the O1 position (Table S2). This host–guest anchoring mechanism accounts for the observed framework adaptation and the resulting elliptical geometry of the 12MR aperture. Conversely, the O4 site exhibits the largest T–O–T angle (163.7°) and lacks direct interactions with the water network, suggesting a state of local structural relaxation (Table S2). The connectivity of the water oligomers is further stabilized by the w2···w1 bridging interaction (3.168 Å) and the w3···w3 distance (3.299 Å), which together maintain the long-range order of the hydration shell across the supercage.

### 2.2. Rietveld Refinements After Drugs Loading

**Figure 2:**
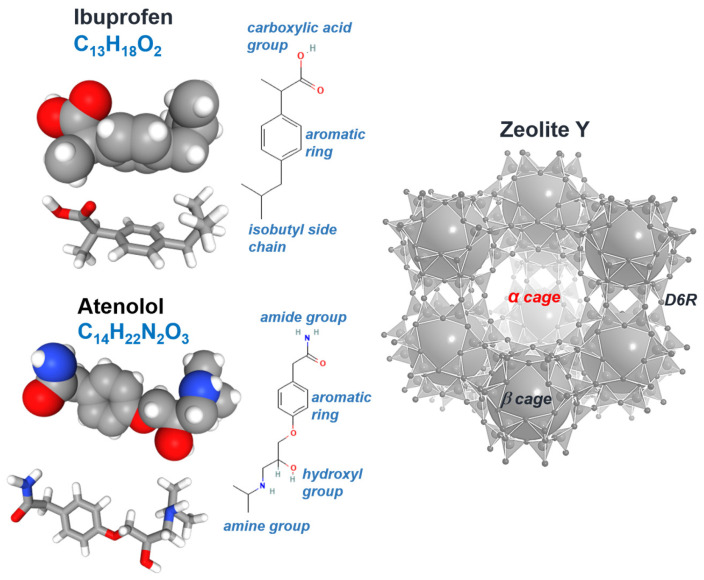
Structures of ibuprofen, atenolol and the framework of Y zeolite. The Y zeolite structure consists of a three-dimensional network of α-cages, β-cages, and double six-ring (D6R) units, forming 12-membered rings (12MR) channels.

Ibuprofen (2-[4-(2-methylpropyl) phenyl] propanoic acid, IBU) is a chiral, amphiphilic molecule composed of three main functional domains: a carboxylic acid group, an aromatic ring, and an isobutyl side chain (Figure 2).

The carboxyl group represents the polar head, capable of forming hydrogen bonds and polar interactions, while the aromatic ring forms a hydrophobic core. The branched isobutyl group acts as a hydrophobic tail, contributing to steric confinement and dispersive interactions within the zeolite pores. Together, these functional elements dictate the orientation, binding mode, and stabilization of ibuprofen upon adsorption in the zeolite framework.

Atenolol (β-[4-(2-hydroxy-3-(isopropylamino)-propoxy) phenyl] acetamide, ATE) is a chiral, moderately polar β_1_-selective adrenergic blocker with four functional regions: an aromatic ring, secondary amine, hydroxyl, and amide moiety (Figure 2). The amide acts as a polar head capable of donating and accepting hydrogen bonds, providing primary anchoring within the zeolite. The aromatic ring forms a rigid hydrophobic core for steric matching, the hydroxyl group adds a polar interaction site, and the secondary amine can be protonated, enhancing electrostatic interactions with the negatively charged framework and/or co-adsorbed water.

For both Y-IBU and Y-ATE, initial refinements in cubic symmetry (space group $Fd\bar{3}$) based on difference Fourier maps identified two distinct extra-framework sites within the supercage, labeled C1 and C2. For Y-IBU, these sites were located at x ≈ 0.481, y ≈ 0.481, z ≈ 0.551 and x ≈ 0.462, y ≈ 0.462, z ≈ 0.605, respectively (Table S3). Their refined occupancy corresponds to 10.56 molecules per unit cell (corresponding to 191.6 mg/g), below the steric maximum of ~16 molecules per unit cell, suggesting that ibuprofen loading is primarily controlled by steric factors. Considering the acidic nature of the H-Y framework and the *pKa* of ibuprofen (~4.5), the molecule was refined (and then modeled) in its neutral COOD form.

Similarly, for Y-ATE, the Fourier maxima appeared at x ≈ 0.477, y ≈ 0.477, z ≈ 0.547 for C1 and x ≈ 0.451, y ≈ 0.451, z ≈ 0.598 for C2 (Table S5). For Y-ATE, the refinements yielded a stable configuration in which atenolol is modeled as a protonated species (ND_3_^+^), consistent with its high proton affinity (*pKa*~9.6). The refined occupancy of 0.12 (11.52 molecules per unit cell, corresponding to 273 mg/g) closely matches the theoretical charge-compensation limit of the framework (~11.89 molecules per unit cell), demonstrating that atenolol uptake is governed by electrostatic neutralization of framework charges. Such significant loading levels confirm that the large α-cages of zeolite Y (approx. 822 Å^3^ each) provide an ideal environment for the confinement of these bulky pharmaceutical molecules.

In both systems, site C1 is positioned near the supercage center and exhibited six equivalent orientations related by the threefold axis along [111]. This fragmented distribution, characterized by a distance of approximately 1.49 Å between C1 and C2, indicated that the guest molecules do not occupy a single fixed position, but are distributed across multiple symmetry-equivalent orientations (Figures S1 and S2).

To account for this, a symmetry-lowering procedure was applied using EXPO2014 [16,17]. The structures were refined in the acentric, F-centered triclinic space group F1, which preserves the face-centered cubic lattice while minimizing the number of independent parameters compared to a standard P1 model. Ab initio refinement of the framework in F1 involved 48 independent Si and 96 independent O positions, yielding a geometry fully consistent with the zeolite Y topology. Le Bail refinements further validated the symmetry reduction to Laue class −1, accompanied by anisotropic lattice distortions induced by guest inclusion. Guest molecules were localized using simulated annealing (SA) treating the framework as rigid and the guest molecules as independent fragments. To maintain an optimal parameter-to-reflection ratio, refinement was limited to the external degrees of freedom (translational/rotational) and selected internal torsions (the phenyl–carboxyl linkage for ibuprofen and the ethanolamine chain for atenolol). This approach allows limited conformational flexibility while preserving intramolecular geometry. The SA cost function combined agreement with neutron diffraction data (R_wp_) and a Lennard–Jones potential to prevent unphysical interatomic contacts.

For Y-IBU, the SA procedure converged reproducibly to a stable solution where the refined molecular positions in F1 symmetry are related by a pseudo-symmetry d-glide plane. The refined lattice parameters determined by Le Bail refinement are a = 24.610 Å, b = 24.325 Å, c = 24.292 Å, with angles α = 90.24°, β = 89.56°, γ = 89.67°, yielding a cell volume of 14,541.0 Å^3^ (Table 1). Molecular geometry is consistent with that of free ibuprofen, with internal bond angles in the range 100–120°. The persistence of this symmetry element from the parent $Fd\bar{3}$ structure indicates that the symmetry reduction reflects an ordered framework distortion induced by guest inclusion, rather than random disorder. Similarly, for Y-ATE, the lattice parameters by Le Bail method (a = 24.609 Å, b = 24.330 Å, c = 24.294 Å, with angles α = 90.22°, β = 89.54°, γ = 89.67°) and an expansion of the unit cell volume from 14,332.7 Å^3^ to 14,545.0 Å^3^ (Table 1), corresponding to an increase of approximately 1.5% reflecting a genuine physical response to the presence of the guest molecule.

Interestingly, while the initial cubic models of the loaded samples suggested a slight contraction, the more accurate triclinic models reveal a significant unit cell expansion Δ ≈ 210 Å^3^), which is a direct consequence of the steric pressure exerted by the pharmaceutical guests within the supercages. The drastic reduction in R_wp_ values (from ~8% to ~3%) further validates that this symmetry breaking is a long-range, cooperative phenomenon induced by the guest molecules. Additionally, the calculated loading capacities (191.6 mg/g for ibuprofen and 273.0 mg/g for atenolol) demonstrate that Zeolite Y is an exceptionally effective sorbent, far surpassing many traditional materials. The nature of this effectiveness, however, differs between the two molecules: in Y-ATE, the loading of 11.52 molecules/u.c. is remarkably close to the framework’s charge compensation limit (~11.89 Al/u.c.). At the experimental *pD* of 9.2, which is close to the *pK_a_* (9.6), a significant fraction of atenolol is protonated. Our results suggest that its adsorption is primarily driven by electrostatic interactions and the neutralization of the framework’s negative charge, leading to a highly stable configuration. In Y-IBU, despite being anionic in the bulk solution (*pD* 6.5 vs. *pK_a_* 4.9), the high-silica environment promotes a loading of 10.56 molecules/u.c. Here, the stabilization is governed by steric confinement and hydrogen-bonding networks between the drug and the co-adsorbed water molecules, as revealed by the neutron scattering density maps.

Overall, the combined use of symmetry-lowered ab initio framework refinement and Simulated Annealing for guest localization provided a consistent and physically meaningful description of the host–guest systems. The refined structures capture both the framework distortion associated with drug inclusion and the distinct loading mechanisms governing ibuprofen and atenolol incorporation.

### 2.3. Drug–Zeolite Interactions: Molecular Anchoring and Framework Adaptation

#### 2.3.1. Ibuprofen–Zeolite Interactions

**Figure 3:**
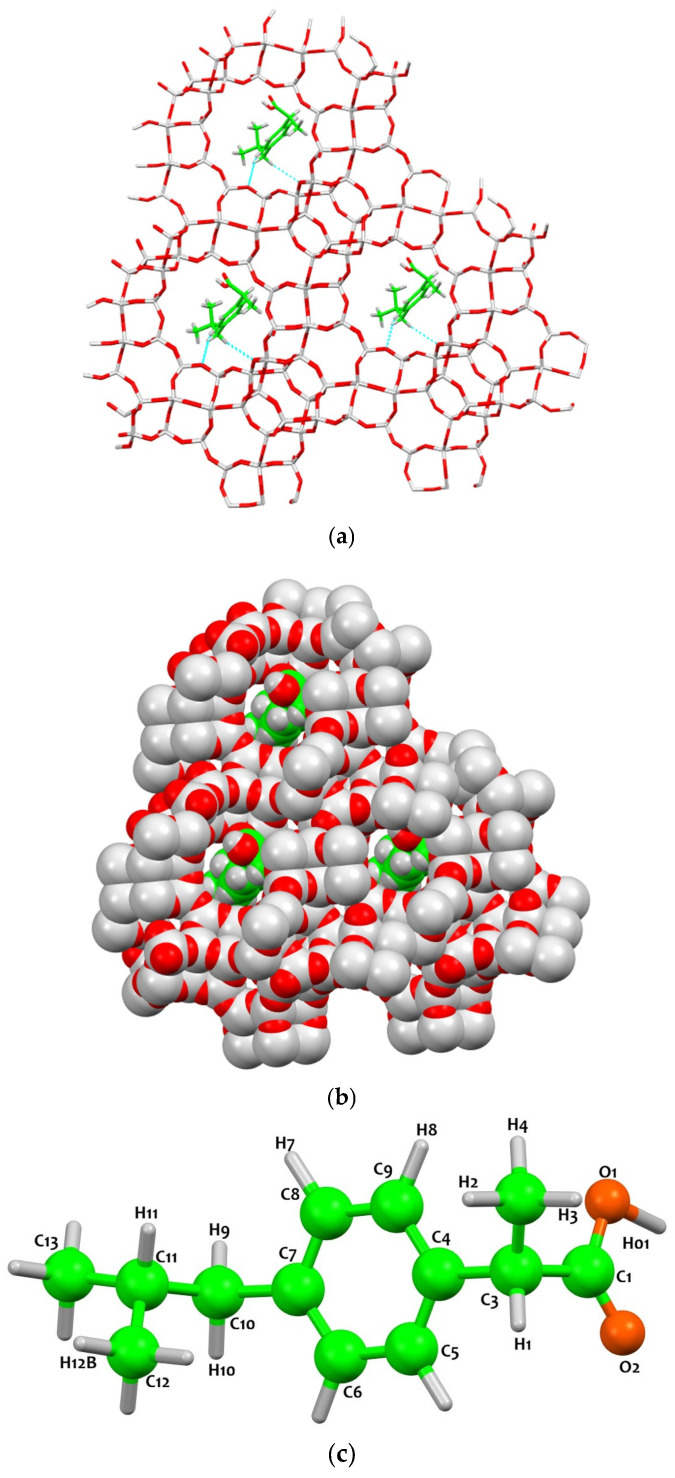
Ab initio models of Y after ibuprofen adsorption (space group F1). (**a**) Perspective view of the framework (grey: Si/Al; red O) with green and orange atoms indicating the C and O sites of ibuprofen. (**b**) Close-up of a single 12MR channel showing drug molecule interaction (O1, O4, C1, C2). (**c**) Labeled molecular structure of ibuprofen.

The molecular geometry and interaction patterns of ibuprofen in various aqueous and solid environments have been extensively investigated [18,19], providing a baseline for our structural models. The specific conformation of the drug within the zeolite is influenced by its intrinsic isomerism [20,21] and the high-level ab initio stability of the molecule [22]. Such structural transitions and the role of supramolecular interactions in defining crystal symmetry are well-documented in the study of organic polymorphisms [23]. The adsorption of ibuprofen within the supercages of Y zeolite was investigated by examining interatomic distances between the drug and the framework. It is important to note that all hydrogen atoms reported in the structural model are, in fact, deuterium, as the ibuprofen loading was performed using a deuterated ibuprofen solution in D_2_O (see Section 3). The data reveal that ibuprofen is stabilized within the supercages through two complementary mechanisms: framework-driven confinement, which dictates the orientation and anchoring points of the molecule, and molecule-driven adaptation, which allows the drug to adjust its conformation to the confined environment (Figure 3).

The isobutyl substituent constitutes the primary framework-imposed anchoring point. The tertiary carbon atom C10 is positioned at 3.368 Å and 3.471 Å from framework oxygens (O2_1-1 and O1_29-1, respectively). Additional contacts involve aliphatic hydrogens, with H10 at 2.685 Å from O1_1-1 and H10A at 2.694 Å from O1_29-1 (Table 2). These distances correspond to near-contact interactions arising from geometric confinement rather than specific directional bonds. As a result, the orientation of the isobutyl group is largely dictated by the supercage topology, which restricts its positional freedom and fixes the aliphatic chain against the internal surface (Figure 3).

The phenyl ring is similarly constrained by the framework, aligning along the cage wall. Carbon C6 lies at 3.419 Å from O1_29-1, and the closest host–guest contact is observed between H6 and O1_29-1 at 2.642 Å (Table 2). This arrangement, driven by the geometry of the supercage and the available void space, limits both rotational and translational motion of the aromatic core, rather than relying on specific hydrogen bonding (Figure 3).

In addition to these framework-imposed interactions, the ibuprofen molecule exhibits a compact intramolecular packing to fit within the supercage. The distance between methyl hydrogen H12 and aromatic proton H8 is 1.914 Å, and between H12 and aromatic carbon C8 is 2.149 Å (Table 2). These short contacts are purely molecule-driven, reflecting the conformational adaptation of the drug to the confined environment (Figure 3). Recently, Gackowski and Paczwa [24] studied ibuprofen in ultra-stable zeolite Y (USY) with a higher SiO_2_/Al_2_O_3_ ratio of 60, H-form, and similar Na_2_O content. In that system, extensive dealumination generates mesopores and extra-framework aluminum (EFAl), which, together with surface hydroxyl groups and water molecules, strongly influence ibuprofen mobility. Hydrogen bonding between the carboxyl group and OH groups or water, as well as coordination to EFAl, contributes significantly to stabilization. The drug remains highly mobile within the mesopores until the loading exceeds approximately 38 wt%, at which point crystallization occurs on the external surface. In contrast, the Y720 zeolite used in the present study has a lower SiO_2_/Al_2_O_3_ ratio (~30) and then reduced dealumination, resulting in preserved microporosity and absence of EFAl species. Consequently, specific hydrogen bonding and coordination interactions are minimal, and stabilization relies predominantly on steric confinement and van der Waals contacts with the framework. Molecule-driven conformational adaptation plays a more prominent role in accommodating ibuprofen within the smaller supercages of Y than in mesoporous USY.

Overall, these results highlight the critical role of framework composition and pore topology in defining host–guest interactions, demonstrating that ibuprofen stabilization can be rationalized as a combination of framework-driven anchoring and molecule-driven conformational adaptation within the confined environment.

#### 2.3.2. Atenolol–Zeolite Interactions: Polar Anchoring and Confinement-Driven Stabilization

The immobilization of atenolol within the HY zeolite framework is a highly coordinated process, often described as a “hand-in-glove” fit. The molecule, a chiral and moderately polar beta-blocker, distributes its four functional regions (the amide moiety, aromatic ring, hydroxyl group, and secondary amine) within the nanoporous environment of the zeolite. The immobilization of atenolol mimics the conformational isomorphism observed between its racemic and homochiral forms [25]. The localization of such guests was performed using reciprocal and real space methods [26]. The specific conformational behavior and the energy landscape of these functional regions, particularly regarding their orientation in confined spaces, are consistent with recent theoretical investigations into the molecular stability and enantiomer migration of atenolol [27,28]. Upon adsorption, the HY framework undergoes a notable structural adaptation to accommodate these moieties.

**Figure 4:**
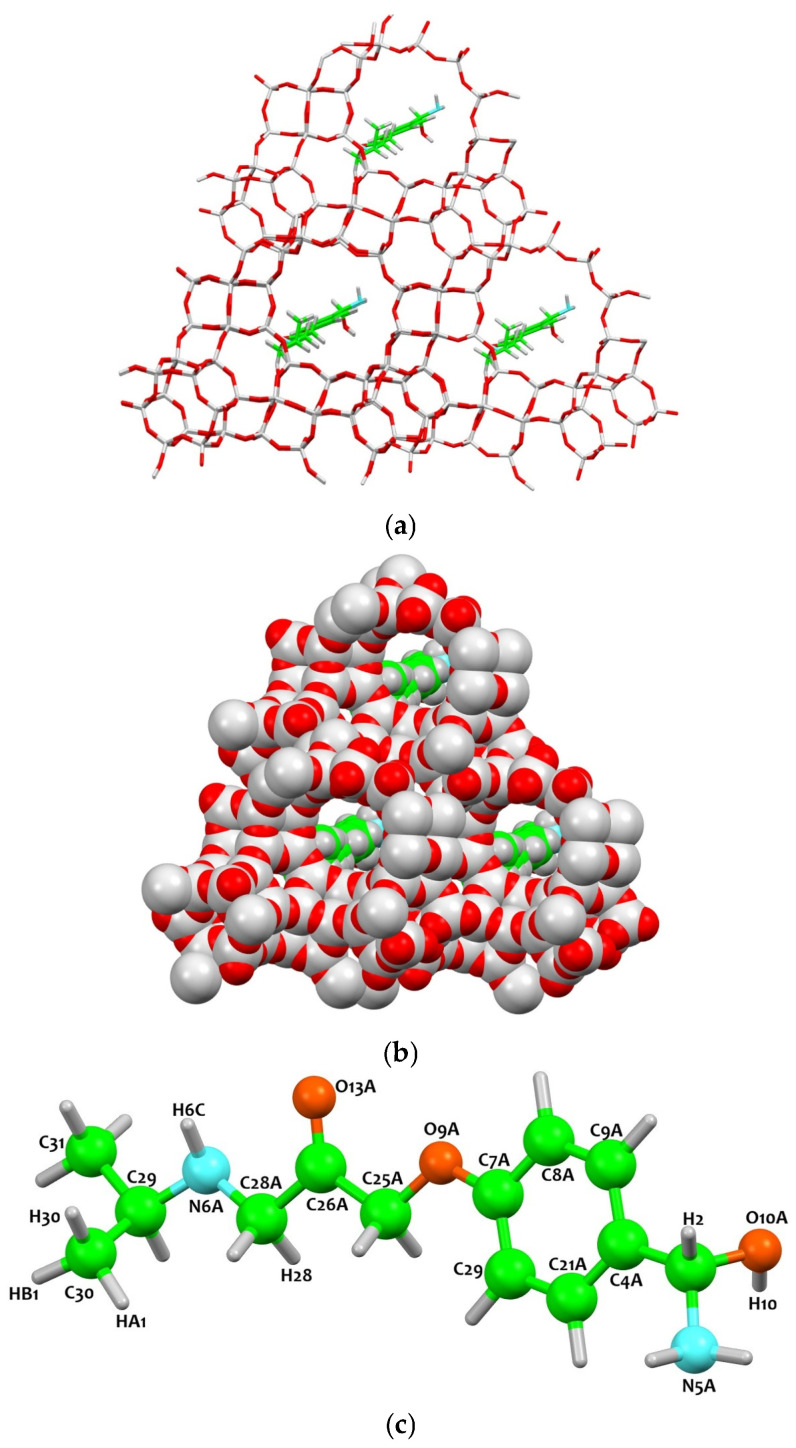
Ab initio models of zeolite Y after atenolol adsorption (space group F1). (**a**) Perspective view shoowing atenolol (green C; light blue N) within the framework (grey: Si/Al; red O). (**b**) Close-up of guest–host interactions at the oxygen sites. (**c**) Labeled molecular structure of atenolol highlighting the carbon skeleton and nitrogen sites.

The stabilization of atenolol within the zeolite arises from a balance between weak polar contacts and strong hydrophobic confinement. This confinement-driven stabilization is achieved through a precise steric match between the atenolol molecule and the internal topology of the FAU supercage. With a molecular length of approximately 13.5 Å (including the flexible tail), atenolol occupies the supercage (11.2 Å) by adopting a folded conformation that maximizes Van der Waals contacts with the curved silicate walls. Accessibility is governed by the critical diameter (the narrowest cross-section, ~5.5–6.5 Å), allowing the molecule to pass through the 7.5 Å apertures via configurational diffusion. Unlike many host–guest systems dominated by rigid hydrogen bonding, atenolol relies heavily on steric matching within the supercages of the zeolite (Figure 4). The polar groups (the secondary amine, hydroxyl, and amide moieties) function as flexible tethers rather than rigid anchors (Figure 4). The secondary amine interacts with framework oxygens O4-1 and O2-4 at a distance of approximately 3.6 Å, and the hydroxyl group forms a similar contact with O2-2 at the same distance (Table 3). These distances exceed those typical of classical hydrogen bonds, indicating that the interactions are primarily weak electrostatic and van der Waals contacts [28], allowing the molecule to adapt to the local chemical environment of the channel walls.

In contrast, the hydrophobic regions of the molecule are tightly constrained within the framework. The physical mechanism of confinement is twofold: first, the 12-membered ring (12MR) windows, with a diameter of ~7.4 Å, act as a kinetic bottleneck, restricting the translational motion of the bulky aromatic core (approx. 6 Å wide). Second, the entropic gain from the displacement of structured water clusters from the supercage provides the thermodynamic driving force that traps the drug in a state of ‘hydrophobic snugness.

The aromatic core is wedged into the channel such that hydrogen H9 interacts with framework oxygen O2-1 at 2.36 Å, complemented by additional CH···O contacts up to 3.26 Å (Table 3). This terminal group acts as a structural ‘anchor’ or lock; because its dimensions are nearly identical to the local curvature of the framework, it prevents the molecule from diffusing through the narrow 12MR windows. This confinement restricts both rotational and translational freedom of the aromatic ring. The strongest confinement is observed at the terminal alkyl group, where carbons C29–C31 and associated hydrogens (H30, HB1) interact with supercage oxygens O1-4, O2-4, O1-3, and O4-1 at distances as short as 2.25 Å, approaching the van der Waals limit (Table 3). In this way, the terminal group acts as a structural lock, firmly anchoring the molecule within the supercage (Figure 4).

Overall, the host–guest interactions reflect a combination of weak polar anchoring and strong steric confinement from the aromatic core and alkyl tail. This dual stabilization mechanism allows the framework to adapt locally to the guest molecule, resulting in a highly stable complex. The dynamic flexibility of the HY zeolite, coupled with the strategic positioning of Atenolol’s functional groups, ensures that the molecule is both chemically tethered and physically wedged within the nanoporous structure, accounting for the robustness of the host–guest assembly regardless of molecular chirality. Refinement under deuterated conditions confirms that the hydrogen-bonding functionalities of the amide, hydroxyl, and secondary amine groups are preserved, matching spectroscopic observations that these functional signatures remain distinct in both racemic and enantiomeric forms of the drug [29,30]. This architecture highlights the cooperative interplay between molecular functionality and framework flexibility, demonstrating that adsorption geometry and stabilization are predominantly dictated by the distribution of functional groups rather than by stereochemistry.

In conclusion, atenolol immobilization within HY zeolite arises from a synergistic combination of weak polar interactions, van der Waals contacts, and steric confinement. These interactions induce subtle yet significant local distortions of the framework, emphasizing the dynamic adaptability of high-silica zeolite Y toward structurally complex pharmaceutical molecules. The adsorption performance observed in this study is highly competitive compared to other zeolite-based systems recently reported in the literature. For instance, recent research on large-pore silicas and dealuminated faujasites has shown that framework engineering is critical for handling multi-drug systems in wastewater [31,32,33,34]. Our high-silica Zeolite Y achieves uptake values of 191.6 mg/g for ibuprofen and 273.0 mg/g for atenolol. These capacities are significantly higher than those reported for many modified natural zeolites [35], highlighting the advantage of the FAU framework’s large supercages and high hydrophobicity. As previously demonstrated for ibuprofen in similar porous architectures [34,36], the adsorption capacity is strongly influenced by the pore volume and the specific host–guest interactions. By resolving the exact anchoring points of these drugs, our study provides the structural evidence needed to justify the high removal efficiencies observed in large-scale environmental applications [33,34,35,36,37].

## 3. Materials and Methods

### 3.1. Materials and Sample Preparation

The starting material utilized in this study was a powdered FAU type zeolite Y (Y720, CBV720) provided by Zeolyst International (Conshohocken, PA, USA), characterized by a SiO_2_/Al_2_O_3_ molar ratio of 30 and a Na_2_O content of 0.03 wt%. Zeolite Y crystallizes in the cubic space group $Fd\bar{3}$ m space group (a ≈ 24.7 Å), and consists of a three-dimensional framework of corner-sharing TO_4_ tetrahedra (T = Si, Al). This topology generates large α-cages with a diameter of approximately 11.2 Å, which are interconnected via β-cages and double six-membered rings (D6Rs) (Figure 1).

To optimize neutron scattering contrast and suppress incoherent background noise from hydrogen, deuterated analogues of the target pharmaceuticals were employed: deuterated atenolol (C_14_D_7_H_15_N_2_O_3_) and deuterated ibuprofen (C_13_D_3_H_15_O_2_). Both reagents were purchased from Sigma-Aldrich (St. Louis, MO, USA) with a certified purity ≥ 99%, and used as received.

### 3.2. Drug Loading Procedure

Adsorption experiments of both pharmaceuticals were performed by batch-loading procedure in deuterated solvent (D_2_O), to promote high uptake and efficient H/D isotopic exchange. For ibuprofen loading, a 0.1 mM solution of deuterated ibuprofen in D_2_O was prepared. Approximately 1.0 g of zeolite powder was added to 70 mL of this solution and magnetically stirred at room temperature (25 ± 0.5 °C) for five days. The measured *pD* of the loading solutions was 6.5 for ibuprofen and 9.2 for atenolol. These values are consistent with the intrinsic acidity/basicity of the solutes. Considering their respective *pK_a_* values (*pK_a_*~4.9; *pK_a_*~9.6), ibuprofen exists as an anionic species in the bulk, while atenolol remains significantly protonated (>70%). The influence of these states on the final observed orientation and anchoring within the zeolite supercages is discussed in Section 2.

To ensure the stability of the zeolite framework during the prolonged stirring process, the unit cell parameters and peak profiles were monitored and compared with the pristine material, serving as an internal structural control.

The solid was then recovered by filtration through 0.22 µm nylon membranes. To maintain the integrity of the H/D exchange and prevent unintended rehydration from atmospheric moisture—a critical control variable for accurate guest localization—all final filtration, rinsing, and drying steps were performed inside a nitrogen-filled glove box. The sample (Y-IBU) was then rinsed with 6 mL of D_2_O, dried on a glass slide under a gentle stream of nitrogen for three days, and loaded into an 8 mm vanadium holder inside the glove box for neutron diffraction analysis. Atenolol loading was performed using a 0.19 mM solution of deuterated atenolol in D_2_O, following the same procedure as described for ibuprofen. After adsorption, filtration, rinsing, and drying under inert atmosphere, the atenolol-loaded sample (Y-ATE) was sealed in an 8 mm vanadium holder under nitrogen atmosphere to ensure data consistency.

### 3.3. Diffraction Experiments

The pristine zeolite Y sample (Y) was firstly characterized by X-ray powder diffraction (XRPD) to establish reference structural parameters. XRPD pattern was collected using a Bruker D8 Advance DaVinci diffractometer (Billerica, MA, USA) operating in Bragg–Brentano geometry, equipped with a Cu Kα X-ray source and a LynxEye XE silicon strip detector. Data were acquired in continuous mode over the 2θ range of 5–90°, with a step size of 0.02° and a counting time of 2 s per step.

Neutron powder diffraction (NPD) experiments were carried out at the Institut Laue-Langevin (ILL, Grenoble, France) using the high-resolution two-axis diffractometer D2B (ILL, Grenoble, France). A Ge (335) monochromator provided a constant-wavelength neutron beam (λ = 1.594 Å). Measurements were performed in Debye–Scherrer geometry to ensure high statistical accuracy and minimize preferred orientation effects. Drug-loaded zeolite samples were sealed in 8 mm diameter vanadium cylinders under nitrogen atmosphere to prevent atmospheric moisture contamination. Neutron diffraction data were collected at 1.5 K over a 2θ range of 4–161°, with a step size of 0.02°. The use of low-temperature collection was a deliberate experimental design choice to minimize thermal vibrations, thereby enhancing the visibility of the guest molecules within the supercages.

### 3.4. Rietveld Structure Refinements

The crystal structure of pristine zeolite Y was refined in the $Fd\bar{3}$ space group via Rietveld analysis using the GSAS software package (Report No. LAUR-86-748, Los Alamos, NM, USA) with the EXPGUI interface [31,32]. The resulting structural parameters from the X-ray powder diffraction (XRPD) data were subsequently employed as the starting model for refining neutron powder diffraction data of the drug-loaded samples (Y-ATE and Y-IBU).

All refinements utilized a pseudo-Voigt peak profile function with a 0.01% intensity cut-off. For XRPD data, profile parameters included two Gaussian terms (GW, GU) and two Lorentzian contributions (LX, LY). Neutron diffraction patterns were fitted using three Gaussian terms (GW, GU, GV), one Lorentzian term (LX), and an asymmetry parameter. Background contributions for all datasets were modeled using an 18-term Chebyshev polynomial, while scale factors and 2θ zero shifts were refined for all datasets. To ensure the reliability and statistical validity of the models, the quality of the fit was monitored through the weighted profile R-factor (R_wp_), the goodness-of-fit (χ^2^), and the flat nature of the difference curve.

Geometrical soft restraints were applied to maintain the framework connectivity: T–O and O–O distances within the TO_4_ tetrahedra were restrained to 1.66 ± 0.04 Å and 2.660 ± 0.040 Å, respectively. These restraints were gradually relaxed in the final stages of refinement, with no significant deviations observed in the interatomic distances. The validation of guest localization was performed via iterative calculation of Difference Fourier (nuclear density) maps. This procedure allowed for the unambiguous identification of residual density peaks corresponding to the pharmaceuticals and co-adsorbed water clusters. To prevent over-parameterization and ensure model stability, isotropic displacement parameters U_iso_ were refined in blocks, constrained by chemical species. The lattice parameters and the space group symmetry for the loaded samples were initially confirmed through Le Bail profile fitting before proceeding with full Rietveld structural refinement.

Convergence was achieved by simultaneous refinement of profile parameters, scale factors, atomic coordinates, site occupancies and U_iso_.

For the guest molecules, soft constraints were applied to typical bond lengths (C–C 1.54 Å, C=C 1.39 Å, C–O 1.43 Å, C=O 1.25 Å, C–D 1.08 Å, O–D 0.96 Å; ±0.04 Å) to preserve realistic molecular geometry. These were progressively relaxed in the final refinement cycles. Convergence was achieved by simultaneously refining profile parameters, scale factors, atomic coordinates, and site occupancies. To ensure model stability and consistency, isotropic displacement parameters were refined in blocks, where a single U_iso_ value was constrained for all atoms of the same chemical species (i.e., Si, O, C, N, and D).

**Figure 5:**
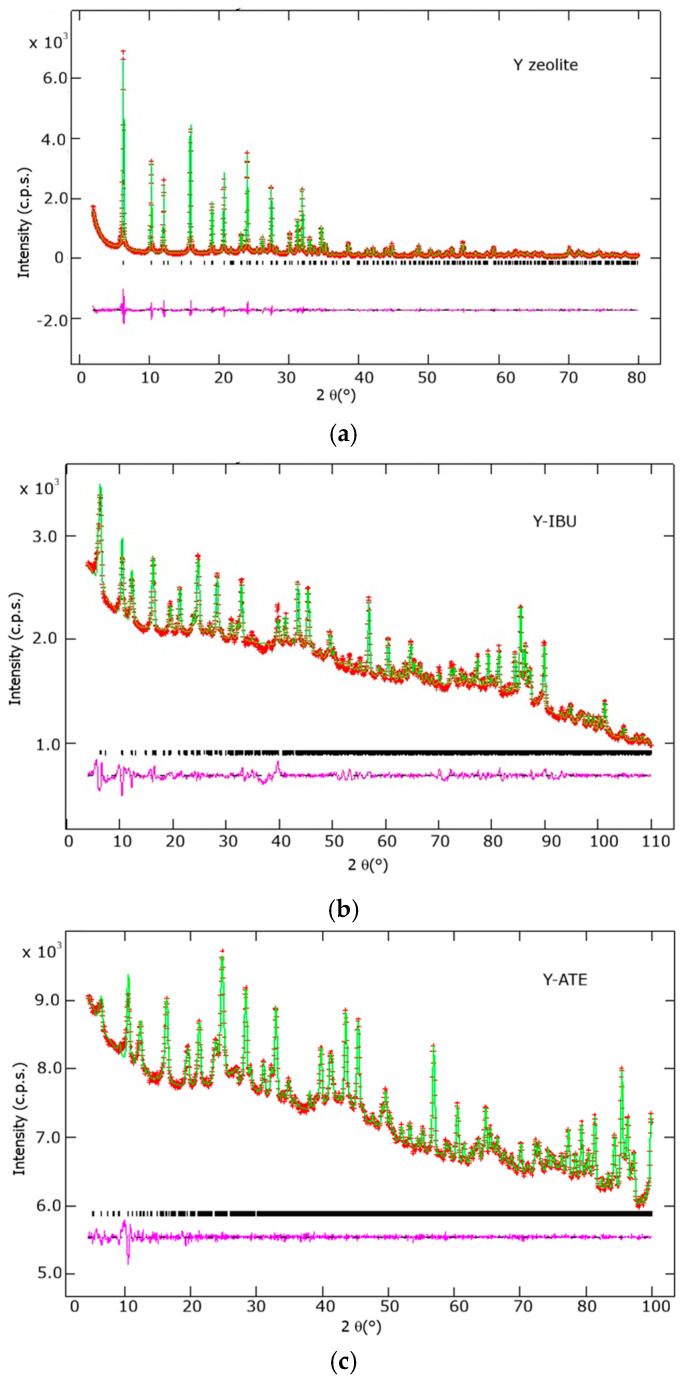
Rietveld refinement patterns for the zeolite samples. Observed data (crosses), calculated model (continuous line), and the difference curve (bottom line) are shown for: (**a**) Pristine Zeolite Y refined from Synchrotron/Laboratory XRD data, serving as the structural baseline; (**b**) Y-IBU and (**c**) Y-ATE refined from Neutron Powder Diffraction (NPD) data after pharmaceutical loading. All initial patterns are displayed in the cubic $Fd\bar{3}$ space group to highlight the high quality of the fit and the framework stability prior to the determination of the guest-induced symmetry reduction.

All hydrogen atoms were treated as deuterium, consistent with sample preparation and neutron refinements were performed against data collected at 1.5 K. The Rietveld refinement plots of the samples collected before and after loading are shown in Figure 5.

Details of the structural refinements are reported in Table 1. Further technical details regarding the crystallographic modeling strategy, the interpretation of electron density maps, and the treatment of average symmetry in drug-loaded samples are provided in the Supplementary Information. Selected bond distances and angles are provided in the Supplementary Information (Tables S1–S6). Crystallographic Information Files (CIFs) for all samples have been deposited as electronic Supplementary Materials.

## 4. Conclusions

This study demonstrates the significant structural adaptability of high-silica zeolite Y upon adsorption of the pharmaceutical molecules ibuprofen and atenolol. By integrating neutron and X-ray diffraction techniques with advanced Rietveld refinement, we have elucidated the precise mechanisms of framework modulation and guest accommodation. The application of neutron diffraction under hydrated and deuterated conditions proved instrumental in visualizing host–guest interactions involving light atoms, offering a comprehensive description of the localized distortions within the aluminosilicate matrix. Even in its pristine hydrated state, the nominally cubic framework ($Fd\bar{3}$) exhibits localized deviations from ideal symmetry. These perturbations arise from the formation of hydrogen bonding networks between framework oxygens and extra-framework water molecules. Our structural investigation, supported by neutron powder diffraction, demonstrates that high-silica zeolite Y is an exceptionally effective sorbent for the removal of common pharmaceuticals from aqueous phases. The achieved loading capacities—191.6 mg/g for ibuprofen and 273.0 mg/g for atenolol—surpass those of many traditional carbon-based materials, highlighting the potential of the FAU-type framework for environmental remediation. Upon the adsorption of pharmaceutical guests, a systematic symmetry reduction from cubic $Fd\bar{3}$ to the acentric, F-centered triclinic symmetry (F1, Laue class −1) is observed. This transition reflects an ordered, cooperative framework response rather than random disorder, preserving the translational characteristics of the parent lattice while accommodating the specific geometries of the asymmetric guest molecules.

The adsorption of ibuprofen induces a moderate anisotropic expansion of the unit cell along with localized pore relaxation. The refined structural model indicates that guest stabilization is governed primarily by steric confinement and dispersive interactions involving the aromatic ring and isobutyl group, which are complemented by weaker polar interactions with the carboxylic group. The refined occupancy remains below the steric maximum, consistent with a loading mechanism controlled predominantly by spatial constraints rather than framework charge compensation.

In contrast, atenolol adsorption results in a more pronounced overall unit cell expansion, while simultaneously imposing tighter local confinement within the supercages. The stabilization of atenolol is achieved through a synergistic combination of polar contacts involving the protonated amine, hydroxyl, and amide groups, alongside strong van der Waals confinement of the aromatic and alkyl segments. The refined occupancy closely corresponds to the theoretical charge compensation limit of the framework, confirming that atenolol uptake is primarily driven by the electrostatic neutralization of framework charges. Importantly, molecular chirality was found to play a secondary role in both systems. Racemic and homochiral species adopt comparable conformations and interaction geometries within the zeolitic cavities, indicating that adsorption behavior is dictated by the spatial distribution and chemical functionality of the guest rather than its stereochemical configuration.

In summary, the results establish that drug adsorption in zeolite Y is a cooperative host–guest process characterized by a guest-induced structural adaptation. This phenomenon involves a transition to a lower symmetry system, anisotropic lattice distortion, and a localized relaxation of the pore geometry to accommodate the pharmaceutical guests. The capacity of the framework to accommodate chemically distinct pharmaceutical molecules while maintaining long-range crystallinity underscores the utility of zeolite Y as a model system for investigating structure–function relationships in adsorption-driven framework modulation.

## Figures and Tables

**Table 1:** Rietveld refinement details and crystallographic parameters for pristine Zeolite Y, IBU-loaded (Y-IBU), and ATE-loaded (Y-ATE) samples. Data for loaded samples were collected at 1.5 K to minimize thermal displacement of guest molecules. R_p_ (Profile R-factor) and R_wp_ (Weighted Profile R-factor) represent the overall agreement between the observed and calculated diffraction profiles; RF^2^ (Structure Factor R-factor) specifically assesses the consistency of the structural model (atomic positions and occupancies) with the experimental intensities. * = Le Bail method.

	Pristine Y ($Fd\bar{3}$)	Y-IBU ($Fd\bar{3}$)	Y-ATE ($Fd\bar{3}$)	Y-IBU (*F*1) *	Y-ATE (*F*1) *
a (Å)	24.291(1)	24.265(3)	24.264(1)	24.610(2)	24.609(1)
b (Å)	24.291(1)	24.265(3)	24.264(1)	24.325(3)	24.330(2)
c (Å)	24.291(1)	24.265(3)	24.264(1)	24.292(3)	24.29(1)
α (°)	90	90	90	90.24(1)	90.22(1)
β (°)	90	90	90	89.56(1)	89.54(1)
γ (°)	90	90	90	89.67(1)	89.67(1)
Cell volume (Å^3^)	14,332.7(5)	14,286.6(1)	14,284.1(5)	14,541.0(3)	14,545.0(2)
No. of variables	68	65	65	28	28
No. of reflections	3920	3618	3118	3118	3618
R_p_ (%)	7.13	7.65	7.89	2.33	1.32
R_wp_ (%)	7.87	7.98	8.20	2.97	1.89
RF^2^ (%)	9.67	9.78	9.97	-	-

**Table 2:** Host–guest interactions and distances (Å) for ibuprofen in zeolite Y.

Functional Region	Framework Atoms	Distance (Å)	Interaction Type
H6 (Aromatic, D)	O1_29	2.642	Weak CH···O/Steric Fit
H10 (Isobutyl, D)	O1_1	2.685	Hydrophobic Anchoring
H10A (Isobutyl, D)	O1_29	2.694	Hydrophobic Anchoring
C10 (Aliphatic)	O2_1	3.368	Steric Confinement
C10 (Aliphatic)	O1_29	3.471	Steric Confinement
C6 (Aromatic)	O1_29	3.419	van der Waals
H12 (Methyl, D)	H8/C8	1.914/2.149	Intramolecular Packing
H12B (Methyl, D)	O2_19	3.30	van der Waals

**Table 3:** Host–guest interactions and distances (Å) for atenolol (ATE-D) in zeolite HY.

Functional Region	Framework Atoms	Distance (Å)	Interaction Type
Secondary amine (N6A–H6C)	O4-1, O2-4	~3.6	Weak electrostatic/van der Waals
Hydroxyl group (O10A–H10)	O2-2	~3.6	Weak polar contact
Aromatic ring (C4A–C9A, C21A, C29A)	O2-1	2.36–3.26	Van der Waals/CH···O contacts
Alkyl terminal (C29–C31, H30, HB1)	O1-4, O2-4, O1-3, O4-1	2.25–3.07	Van der Waals contacts

## Data Availability

The data presented in this study are available on request from the corresponding author. The neutron diffraction data were generated at the Institut Laue-Langevin (ILL).

## Supplementary Materials

The following supporting information can be downloaded at https://www.mdpi.com/article/10.3390/molecules31020384/s1, Table S1. Atomic coordinates for the as-synthesized Zeolite Y ($Fd\bar{3}$); Table S2. Framework bond distances (Å) and angles (°) for the as-synthesized Zeolite Y ($Fd\bar{3}$); Table S3. Atomic coordinates of Zeolite Y loaded with atenolol ($Fd\bar{3}$); Table S4. Framework bond distances (Å) and angles (°) for Zeolite Y loaded with atenolol ($Fd\bar{3}$); Table S5. Atomic coordinates for Zeolite Y loaded with ibuprofen ($Fd\bar{3}$); Table S6. Framework bond distances (Å) and angles (°) for Zeolite Y loaded with ibuprofen ($Fd\bar{3}$). Figure S1. (a) 3D view of zeolite Y (grey: Si/Al; red: O) with orange C sites occupied by atenolol molecules. (b) 12MR cross-section highlighting atenolol orientation and proximity to framework oxygen sites (O1, O4). Figure S2. (a) 3D framework view of zeolite Y (grey: Si/Al; red: O) with green C sites occupied by ibuprofen within the 12MR channels. (b) 12MR cross-section showing ibuprofen orientation and host–guest interactions with O1 and O4.
