# Supplementary material for: Combined Neutron and X-Ray Diffraction Study of Ibuprofen and Atenolol Adsorption in Zeolite Y
Source: Molecules. 2026 Jan 22;31(2):384. doi: 10.3390/molecules31020384 (PMC12844287; doi:10.3390/molecules31020384)
Supplement: Supplementary file 1 [file molecules-31-00384-s001.zip › molecules-4100267-supplementary.pdf]

Supplementary Information

Combined Neutron and X-Ray Diffraction Study of Ibuprofen and Atenolol Adsorption in Zeolite Y

Annalisa Martucci [1,*], Maura Mancinelli [1], Tatiana Chenet [2], Luca Adami [1], Caterina D'anna [2], Emmanuelle Suard [3] and Luisa Pasti [2]

[1] Department of Physics and Earth Sciences, University of Ferrara, via Saragat 1, I-44121 Ferrara, Italy; luca.adami@unife.it (L.A.)
[2] Department of Environmental and Prevention Sciences, University of Ferrara, via L. Borsari 46, I-44121 Ferrara, Italy; tatiana.chenet@unife.it (T.C.); caterina.danna@unife.it (C.D.);luisa.pasti@unife.it (L.P.)
[3] Institute Max von Laue and Paul Langevin, D2B Beamline BP156, 38042 Grenoble, France; suard@ill.fr
* Correspondence: mrs@unife.it; Tel.: +39-0532-974767

S1. Crystal Structure Solution and Refinement Strategy (EXPO2014)

Additional Methodological Details for Structure Refinement

1. Space Group Symmetry and Average Structure Treatment

The powder diffraction patterns of the drug-loaded samples were indexed and refined in the cubic Fd-3 space group. We explicitly clarify that, although the presence of ibuprofen or atenolol molecules within the supercages locally breaks the high cubic symmetry (effectively lowering it to P1 or F1 at the level of an individual cage), the long-range periodicity of the FAU framework remains cubic.

In powder diffraction, the measured signal represents a spatial and temporal average over millions of unit cells. As a consequence, the system is correctly described as an average structure. The guest molecules were therefore modeled as disordered electron density distributed over symmetry-equivalent positions within the supercage. This approach is widely accepted and routinely applied to host–guest systems in zeolite science when powder diffraction data are used.

2. Identification of Extra-Framework Sites

The localization of guest molecules was performed using iterative difference Fourier (electron density) maps. The sites labeled C1 and C2, separated by approximately 1.49 angstrom, correspond to centroids of residual electron density. This short inter-site distance is a characteristic crystallographic signature of positional and orientational disorder of the pharmaceutical molecule confined within the supercage. These sites do not represent individual atoms or distinct molecules. Instead, they describe preferred locations of the most bulky functional groups of the drug as it adopts slightly different configurations or dynamically shuttles within the approximately 13 angstrom supercage.

The use of these sites allows a stable refinement of the total guest occupancy while avoiding over-parameterization of the structural model.

3. Molecular Diffusion and Steric Constraints

To assess the feasibility of drug entry into the FAU framework, the critical diameter of the molecules, defined as their smallest lateral cross-section, was considered rather than their maximum longitudinal length (11.2 angstrom for ibuprofen). According to the principles of configurational diffusion and shape selectivity, elongated and flexible molecules such as ibuprofen and atenolol can align their narrowest dimension,

estimated to be approximately 5.5 to 6.5 angstrom, with the 12-membered ring apertures of the zeolite, which have an effective diameter of about 7.5 angstrom. Once inside the pore system, the three-dimensional channel network and the large supercages (approximately 13 angstrom in diameter) allow molecular reorientation and accommodation. This mechanism explains the high loading values observed and confirms that the molecules are not sterically excluded from the internal pore volume.

4. Refinement Stability and Geometrical Restraints

To ensure physical consistency during Rietveld refinement, soft geometrical restraints were applied to the guest molecules, which were treated as rigid bodies during the initial refinement stages. Bond lengths involving carbon, oxygen, and nitrogen atoms were restrained to standard IUPAC values with a tolerance of plus or minus 0.04 angstrom. As refinement convergence was approached, these restraints were progressively relaxed. The stability of the final weighted profile R-factor and chi-squared values, together with the chemical plausibility of the host–guest distances, confirms the validity of the refined structural models.

EXPO2014 Structure Solution Procedure

The crystal structure solution of drug-loaded zeolite Y samples was carried out using the EXPO2014 software suite. Given the inherent challenges of powder diffraction data, such as peak overlap and background ambiguity, a multi-step probabilistic and real-space approach was adopted.

1. **Space Group Determination**
   The extinction symbol and space group were identified using the probabilistic procedures implemented in EXPO2014. This method evaluates the statistical distribution of normalized intensities extracted by the Le Bail method to determine the most probable extinction symbol compatible with the cubic crystal system. Although guest adsorption locally breaks the Fd-3 symmetry, the long-range periodicity of the zeolite framework remains dominant, and the average structure was therefore refined in the Fd-3 space group.
2. **Phase Recovery and E-map Calculation**
   Phase information was recovered using Direct Methods. After extraction and normalization of integrated intensities, triplet invariants were estimated and phases were obtained via the tangent formula. The resulting E-map, constructed using normalized structure factor moduli, provided the initial structural model.
3. **Site Identification and Model Optimization**
   Extra-framework sites (C1 and C2) were identified as maxima in the residual electron density of difference Fourier maps. Due to their close proximity, these sites were interpreted as manifestations of positional disorder rather than discrete atomic positions. Model improvement was achieved through Fourier recycling combined with the Resolution Bias Correction algorithm, which is specifically designed to optimize organic–inorganic structural models derived from powder diffraction data.
4. **Refinement of Guest Molecules**
   Ibuprofen and atenolol molecules were treated as rigid bodies during the final stages of Rietveld refinement. This approach accounts for both steric bulk and configurational diffusion effects, ensuring that the refined positions within the approximately 13 angstrom supercages are physically consistent with the approximately 7.5 angstrom channel apertures. All occupancy factors were determined from the integrated electron density, with constraints applied only where required by crystallographic site symmetry.

Table S1. Atomic coordinates for the as-synthesized Zeolite Y ($Fd\overline{3}$).

Atom	x/a	y/b	z/c	Ui/Ue*100	Fraction

*T*1	-0.0552(3)	0.1250(3)	0.0355(3)	0.25(1)	1.00
*T*2	-0.0537(4)	0.0349(3)	0.1207(3)	0.25(1)	1.00
O1	-0.1087(4)	0.0007(3)	0.1087(3)	0.43(1)	1.00
O2	-0.0055(4)	-0.0057(3)	0.1390(3)	0.43(1)	1.00
O3	-0.0373(3)	0.0696(3)	0.0670(3)	0.43(1)	1.00
O4	-0.0633(3)	0.0776(4)	0.1694(3)	0.43(1)	1.00
W1	0.375000	0.3750(4)	0.3750(3)	8.11(1)	0.32(1)
W2	0.2632(4)	0.3456(4)	0.3146(3)	8.11(1)	1.00(1)
W3	0.4241(4)	0.0349(4)	0.1207(3)	8.11(1)	1.00(1)

Table S2. Framework bond distances (Å) and angles (°) for the as-synthesized Zeolite Y ($Fd\overline{3}$).

T1-O1	1.617(5)	T2-O1	1.601(4)
T1-O2	1.600(6)	T2-O2	1.593(4)
T1-O3	1.608(4)	T2-O3	1.603(4)
T1-O4	1.600(4)	T2-O4	1.599(4)
T1-O1-T2	129.3(2)	T1-O2-T2	149.8(2)
T1-O3-T2	139.4(2)	T1-O4-T2	163.7(2)

Table S3. Atomic coordinates of Zeolite Y loaded with atenolol ($Fd\overline{3}$).

**Atom**	**x/a**	**y/b**	**z/c**	**Ui/Ue*100**	**Fraction**
T1	-0.0613(6)	0.1192(5)	0.0409(5)	0.58(1)	1.00
T2	-0.0490(5)	0.0353(5)	0.1171(5)	0.58(1)	1.00
O1	-0.1058(4)	-0.0022(4)	0.1113(5)	0.61(1)	1.00
O2	-0.0021(1)	-0.0087(4)	0.1315(5)	0.61(1)	1.00
O3	-0.0338(1)	0.0623(4)	0.0588(5)	0.61(1)	1.00
O4	-0.0525(7)	0.0847(1)	0.1606(1)	0.61(1)	1.00
C1	0.4766(2)	0.4766(2)	0.5468(3)	1.86(2)	0.11(5)
C2	0.4511(3)	0.4511(3)	0.5978(3)	1.86(2)	0.11(5)

Table S4. Framework bond distances (Å) and angles (°) for Zeolite Y loaded with atenolol ($Fd\overline{3}$).

T1-O1	1.567(5)	T2-O1	1.658(4)
T1-O2	1.674(6)	T2-O2	1.600(4)
T1-O3	1.594(4)	T2-O3	1.603(4)
T1-O4	1.638(4)	T2-O4	1.598(4)
T1-O1-T2	124.5(2)	T1-O2-T2	119.9(2)
T1-O3-T2	156.0(2)	T1-O4-T2	173.3(2)

Table S5. Atomic coordinates for Zeolite Y loaded with ibuprofen ($Fd\overline{3}$).

Atom	x/a	y/b	z/c	Ui/Ue*100	Fraction
T1	-0.0539(6)	0.1223(5)	0.0356 (5)	0.58(1)	1.00
T2	-0.0546(5)	0.0365(5)	0.1283(5)	0.58(1)	1.00
O1	-0.1055(4)	-0.0001(4)	0.1070(5)	0.61(1)	1.00
O2	-0.0040(1)	-0.0043(4)	0.1413(5)	0.61(1)	1.00
O3	-0.0357(1)	0.0748(4)	0.0781(5)	0.61(1)	1.00
O4	-0.0699(7)	0.0729 (1)	0.1816(1)	0.61(1)	1.00
C1	0.4807(2)	0.4779(2)	0.5510 (3)	1.86(2)	0.11(5)
C2	0.4615(3)	0.4566(3)	0.6053(3)	1.86(2)	0.11(5)

Table S6. Framework bond distances (Å) and angles (°) for Zeolite Y loaded with ibuprofen ($Fd\overline{3}$).

T1-O1	1.607(5)	T2-O1	1.602(4)
T1-O2	1.607(6)	T2-O2	1.607(4)
T1-O3	1.607(4)	T2-O3	1.600(4)
T1-O4	1.598(4)	T2-O4	1.610(4)
T1-O1-T2	138.7(2)	T1-O2-T2	145.7(2)
T1-O3-T2	149.5(2)	T1-O4-T2	143.2(2)

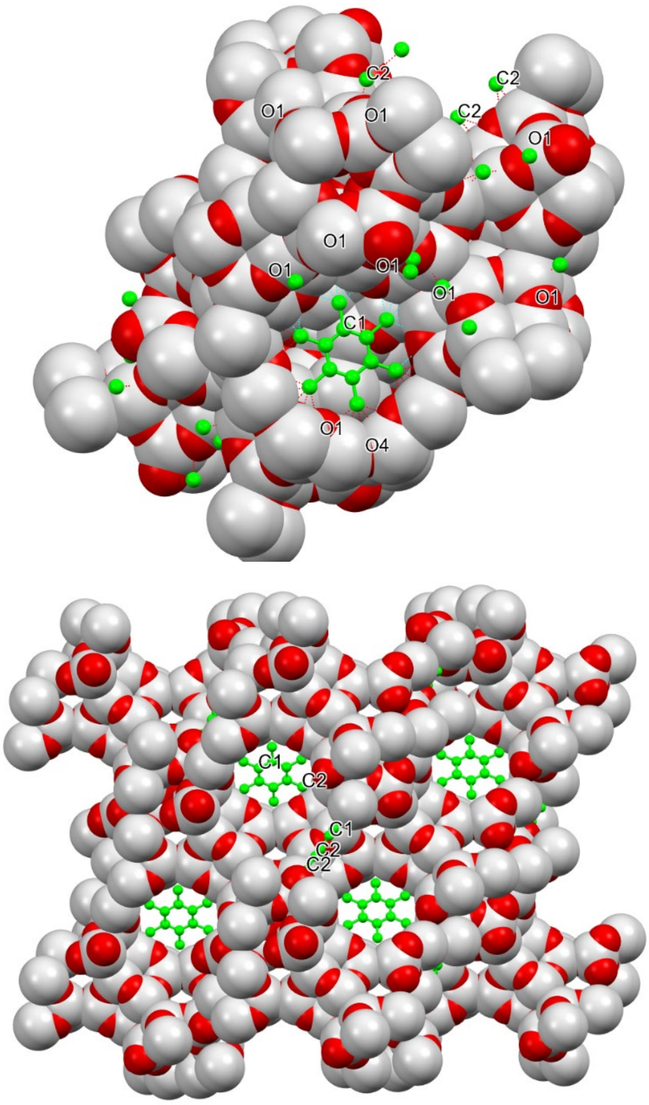

(b)

Figure S1. (a) 3D view of zeolite Y (grey: Si/Al; red: O) with orange C sites occupied by atenolol molecules. (b) 12MR cross-section highlighting atenolol orientation and proximity to framework oxygen sites (O1, O4).

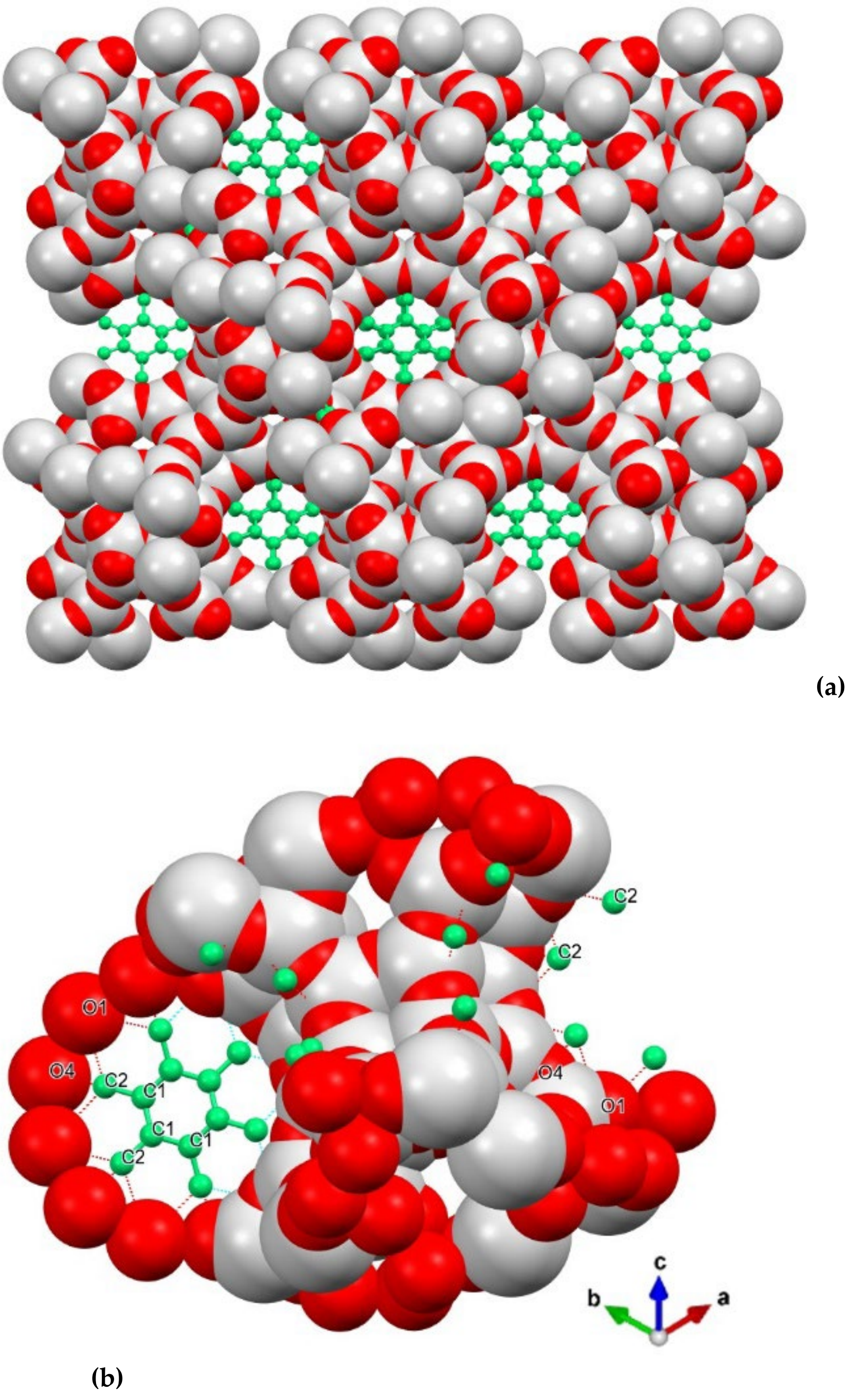

Figure S2. (a) 3D framework view of zeolite Y (grey: Si/Al; red: O) with green C sites occupied by ibuprofen within the 12MR channels. (b) 12MR cross-section showing ibuprofen orientation and host-guest interactions with O1and O4.
